# Molecular Mechanism Analysis of the Effect of Hederagenin Combined with L-OHP on Chemosensitivity of AGS/L-OHP based on Network Pharmacology

**DOI:** 10.2174/0115734099270389240104050955

**Published:** 2024-01-11

**Authors:** Hongyue Tang, Chao Wang, Chenhao Xing, Guoxin Liang, Chang Guo, Xin Liu, YanJie Li, Mingming Zhang

**Affiliations:** 1 Graduate School of Hebei North University, 075000, Zhangjiakou, Hebei, China;; 2 Department of Clinical Medical Research Center, Hebei General Hospital, 050051, Shijiazhuang, Hebei, China;; 3 Hebei Key Laboratory of Metabolic Diseases, Hebei General Hospital, 050051, Shijiazhuang, Hebei, China;; 4 Graduate School of North China University of Science and Technology, 063000, Tangshan, China;; 5 Graduate School of Hebei Medical University, 050051, Shijiazhuang, Hebei, China

**Keywords:** Gastric cancer, hederagenin, drug resistance, chemosensitivity, oxaliplatin, signaling pathway

## Abstract

**Aims and Objectives:**

This study aimed to evaluate the pharmacological mechanism of Hederagenin (HD) combined with oxaliplatin (L-OHP) in treating gastric cancer (GC) through network pharmacology combined with experimental verification.

**Material and Methods:**

Network pharmacology methods were used to screen potential targets for HD, L-OHP, and GC-related targets from public databases, and the intersection of the three gene sets was taken. Cross genes were analyzed through protein-protein interaction (PPI) networks to predict core targets, and related pathways were predicted through GO and KEGG enrichment analysis. The experimental results were verified by the *in vitro* experiments. HD was applied on AGS/L-OHP cells, and then cellular chemosensitivity and the expressions of P-gp, Survivin, Bcl-2, p-Akt, and p-PI3K genes were detected. Wound assay and Transwell Chamber assay were employed to detect the effect of HD on AGS/L-OHP cells. Nude mice xenograft models transfected using AGS/L-OHP cells were also treated with HD in order to verify the results. The size and weight of the tumor, as well as the expressions of P-gp, Survivin, Bcl-2, p-Akt and p-PI3K genes, were also measured.

**Results:**

KEGG analysis showed that the anti-gastric cancer effect of HD was mediated mainly by PI3K-Akt signaling pathways. The PI3K-Akt signaling pathway containing more enriched genes may play a greater role in anti-gastric cancer. It was observed that for AGS/L-OHP cells jointly treated with HD and L-OHP, their activity, migration and invasion were significantly lower than those treated only using HD or L-OHP group. Moreover, expressions of p-Akt, p-PI3K, Bcl-2, P-gp, and Survivin for the HD+L-OHP group decreased significantly. Results of the *in vivo* experiments showed that the sizes and weights of tumors in the HD+L-OHP group were the lowest compared to the HD group and L-OHP group.

**Conclusion:**

Our findings suggest that HD may reduce the resistance of AGS/L-OHP cells to L-OHP by regulating the PI3K/Akt signaling pathway.

## 1 INTRODUCTION

Gastric cancer (GC) is the most common gastrointestinal cancer worldwide [1]. This malignancy is a challenge to be eradicated by surgical operation since most diagnosed patients have already progressed to the advanced stage. Chemotherapy is a crucial part of the comprehensive treatment for GC, despite the fact that the potential drug resistance may reduce the therapeutic efficacy [2, 3]. Oxaliplatin (L-OHP) is a third-generation platinum-based antineoplastic drug, which has been widely used for GC treatment because of its excellent performance than many other chemotherapeutic drugs, such as cisplatin. However, previous studies have reported that L-OHP shows drug resistance [4].

The drug resistance of cancer cells is mediated by the comprehensive regulation of a variety of genes *via* various signaling pathways [5]. The PI3K/Akt signal pathway is involved in a variety of signal pathways that regulate cell differentiation, proliferation, survival, migration, and angiogenesis. Research has reported that the PI3K/Akt signaling pathway is related to drug-resistance treatment of gastric cancer, breast cancer, ovarian cancer, *etc*. [6-9].

Hederagenin is a pentacyclic triterpenoid compound [10] that exists in medicinal plants, such as Hedera helix, Fructus Akebiae, Cyclocarya paliurus, and Sapindus saponaria L [11]. The latest research has reported that it can exert anti-tumor [12], anti-inflammatory [13], and anti-depression effects [14]. Wang *et al.* [15] suggested that ivy saponins can be used as an adjuvant to improve the sensitivity of tumor cells to platinum-based chemotherapy. However, it remains unclear whether HD can change the resistance of GC cells to L-OHP.

In this study, we detected the effect of HD on L-OHP-resistant GC cell lines (*i.e*., AGS/L-OHP) and nude mice transplanted subcutaneously with AGS/L-OHP cells. By measuring the sensitivity of cancer cells to L-OHP and the expressions of drug resistance-related genes in both *in vitro* and animal samples, we aimed to clarify the effect of HD on GC cells with L-OHP resistance and its possible mechanism.

## 2 MATERIALS AND METHODS

### 2.1 Data Preparation

Data for this study were obtained from the following databases:

Database Web link

DAVID https://david.ncifcrf.gov

PharmMapper http://www.lilab-ecust.cn/pharmmapper/index.html

TCMSP tcmspw.com/tcmsp.php

PubChem pubchem.ncbi.nlm.nih.gov/

OMIM omim.org/about

Swiss Target Prediction www.swisstargetprediction.ch/

TTD db.idrblab.net/ttd/

GeneCards www.genecards.org/

UniProt www.uniprot.org/uniprot/

STRING string-db.org/

VENNY2.1.0 https://bioinfogp.cnb.csic.es/tools/venny/

Bioinformatics http://www.bioinformatics.com.cn/

CytoScape3.9.0 software was used in this study.

### 2.2 Screening and Target Prediction

GeneCards database was used to search the L-OHP-related target by searching “oxaliplatin” (with a relevance score greater than 5). CAS numbers and chemical SMILE structure formulas of HD were obtained from the PubChem database. The Drug targets were obtained in Swiss Target Prediction, pharmmapper, HERB, and TCMSP. The effective targets predicted by Swiss Target Prediction were identified using a P-value >0.1 All HD target genes were deduplicated after UniProt correction.

### 2.3 Screening of Disease Targets and Mapping with Drug Targets

The disease targets were obtained by searching “gastric cancer” as keywords in OMIM gene map, TTD, and GeneCards. All GC target genes were deduplicated, and after UniProt correction, the species were selected as “*Homo sapiens,*” and the verified genes (reviewed) were selected to obtain the GC-related target. Subsequently, using VENNY 2.1.0, all targets were mapped to obtain the intersection targets.

### 2.4 Protein-protein Interaction (PPI) Network and Topology Analyses

The resulting intersection genes were imported into the “multiple proteins” function of the STRING database. The “Homo sapiens” species was selected to construct the PPI network diagram. The “string. interactions. tsv” data of PPI were imported into the CytoScape 3.9.0 software and core target mining using the Cytohubba plugin. The three parameters were set to betweenness, Maximum Neighborhood Component (MNC), and Maximal Clique Centrality (MCC), which were selected for screening core targets of HD plus L-OHP-based chemotherapy used in GC.

### 2.5 Gene Ontology (GO) Enrichment and Kyoto Encyclopedia of Genes and Genomes (KEGG) Pathway Enrichment Analyses

The DAVID database (https://david.ncifcrf.gov/) was used for gene ontology (GO) enrichment and enrichment of Kyoto *via* genes and genomes (KEGG). Go enrichment includes biological processes (BP), cellular components (CC), and molecular functions (MF).

### 2.6 Samples and Other Materials

Between August, 2018 and December, 2019, 30 patients with advanced GC were recruited from the Hebei General Hospital. These patients included 19 males and 11 females (mean age: 62.8±11.8 years), and no one opted for chemotherapy before the surgical operation. For each patient, two copies of cancer and adjacent normal tissues (>5 cm away from the edge of the tumor and without cancer cells) were sampled in the size of 1.0 cm×0.5 cm×0.5 cm, respectively. *In vitro* gross total resection of the tumor was performed, the blood and envelope were removed on a qualitative filter paper, and the tissue was chopped with a small amount of PBS using ophthalmic scissors. The tissue was then digested, and the cells isolated, centrifuged, and resuspended in the RPMI 1640 culture medium for the 3-(4,5-Dimethylthiazol-2-yl)-2, 5-diphenyltetrazolium bromide (MTT) assay. Another copy of the tissues was stored under the -80°C environment for real-time PCR and Western blot test.

### 2.7 Human GC Cell Lines (MKN74, SGC7901, MGC803, and AGS) and the Normal Gastric Epithelial Cell Line (GES-1)

Human GC cell lines (MKN74, SGC7901, MGC803, and AGS) and the normal gastric epithelial cell line (GES-1) were cultured by generation in the research center of the Hebei General Hospital. The cell line resistant to L-OHP, *i.e*., AGS/L-OHP (resistance index: 6.25), was also cultured in our research center. Trizol reagents were purchased from Invitrogen, USA. RT-PCR and real-time RT-PCR kits were obtained from Promega, USA. PCR primers were synthesized by Shanghai Biological Engineering Co., Ltd. Protein extraction kits were purchased from Biorad, USA. Rabbit-anti-human PI3K(#4249), p-PI3K, Akt(#4691), and p-Akt(#4060) antibodies were purchased from Cell Signaling Technology, USA. Rabbit-anti-human MDR1/P-gp(#13342) Survivin(#2808), TOPOIIα (#12286), and GAPDH(#5174) were purchased from Cell Signaling Technology, and mouse-anti-human antibody Bcl-2(sc-7382) and GST-π(SC-53909) were purchased from Santa Cruz, USA. MTT, RPMI 1640 culture medium and HD (purity≥ 98%, CAS 465-99-6) were purchased from Desite Biology, China. L-OHP was purchased from Hengrui Medicine Co., China (500mg/ampoule, lot number: H20000337). BALB/c nude mice were purchased from Huafukang Biotechnology Co., China. All mice were males, aged 4-5 weeks old, and weighed 17.5-23.5 g.

### 2.8 Cell Culture

MKN74, SGC7901, MGC803 AGS, and GES-1 cells were cultured in RPMI 1640 culture medium with 10% fetal calf serum (FCS) and 1% penicillin/streptomycin under an environment of 37°C and 5% CO_2_. AGS/L-OHP cells were cultured with L-OHP (2µg/mL) to maintain the drug resistance, and then the L-OHP was removed 1 week prior to experiments. The cells at the logarithmic growth phase were used for experiments.

### 2.9 Cellular Activity

MTT assay was applied to detect cellular survival. Briefly, single-cell suspensions (5×104 cells/mL) were prepared using GC tissues and adjacent normal tissues sampled from patients and the cultured cell lines, respectively. For every 6 wells (one group) of the single cell suspension, HD (10 µmol/L), HD (10 µmol/L) +L-OHP (5 mg/L) or saline (L-OHP group) were added after the fusion growth of cells reaching 60-70%. When cell culture for 48 h, 20 μL MTT (5 mg/mL) was added to each well. The culture medium was discarded 4 hours later, and then 150 μL of dimethyl sulfoxide was added to each well for another 15 minutes of shaking cultivation. Absorbance (A) values at a wavelength of 490 nm were measured.

## 3 MIGRATION AND INVASION OF GC CELLS 

A wound assay was used to detect the migration of GC cells. Briefly, each single cell suspension (5×10^5^ cells/mL) was inoculated on 6-well plates overnight, after which the bottoms of wells were scratched (vertical to the line) using 200 μL pipette tips. Plates were rinsed using PBS for three times. Serum-free medium was then added and cultured for 48 hours under an environment of 37°C and 5% CO_2_. Experiments were then performed similarly to the MTT assay, coped with HD (10 µmol/L), HD (10 µmol/L) +L-OHP (5 mg/L) or saline. The widths of scratches were pictured before and 24 hours after the experiments. The experiments were repeated three times.

Cell invasion experiments were performed as follows: Transwell chambers were placed in 24-well plates, with the upper and lower chambers separated using the polycarbonate membrane (pore size: 8μm). Afterward, 20 μL Matrigel (1mg/mL) was added to the bottom of each upper chamber. Single-cell suspensions (1×10^5^ cells/mL) were prepared using RPMI 1640 culture medium (without FCS), with 200 μL inoculated into the upper chamber. Then, 600 μL RPMI 1640 medium (containing 10% CFS) was added into the lower chamber. After 16-20 hours of cultivation under an environment of 37°C and 5% CO_2_, Matrigel and cells on the upper chamber were wiped off. The membranes were fixed using 4% methanol for 10 min. After staining with crystal violet, the number of cells in five visual fields was counted randomly using a microscope in 400×. The experiments were repeated three times.

### 3.1 MRNA Expression of MDR1, Survivin, GST-π, TOPOIIα, and Bcl-2

Total RNA was extracted from samples using Trizol reagent and then reversely transcribed to cDNA according to the manufacturer’s instructions. Real-time PCR was applied to quantify the expressions of genes with GAPDH served as an internal reference gene. The PCR system (20μL) followed the manufacturer’s instructions, including 2μL cDNA, 10μL SYBR Green Mix, and 0.5μL forward/backward primers (10 μmol/L). The PCR reaction was 95°C for 5min, then 35 cycles of denaturation at 94°C for 30s, annealing at 60°C for 30s, and extending at 72°C for 60s. Primers were designed using Primer 5.0. The primer sequences were as follows: PCR products were detected using the 1.5% agarose gel electrophoresis. The expression of the target gene was calculated by 2^-ΔΔCt^.

### 3.2 PI3K, Akt, p-PI3K, p-Akt, P-gp, Survivin, GST-π, TOPOIIα, and Bcl-2 Proteins

Western blot method was used to detect PI3K, Akt, P-gp, Survivin, GST-π, TOPOIIα, and Bcl-2 proteins. The total proteins were extracted from the cultured cell lines. For each group, 60 μg proteins were separated by 12% polyacrylamide sodium salt (SDS) gel and then electro-transferred to a PVDF membrane. The membrane was incubated with 5% non-fat milk (prepared using TBST) at room temperature for one hour and then incubated with the primary antibody of the targeted protein or GAPDH primary antibody (as the internal reference) overnight at 4°C. The membrane was then washed using TBST three times, followed by the incubation using the secondary antibody (marked with horseradish peroxidase) at room temperature for one hour. The strips were incubated, exposed, and developed by referring to the electrochemistry (ECL) luminescent solution instructions. Image J analysis was carried out for strip gray value.

### 3.3 Nude Mice Model Translated with AGS/L-OHP Cells

To prepare for 15 xenograft tumor models, 2×10^6^ AGS/L-OHP cells were injected subcutaneously into nude mice and allowed to grow for 3 weeks when the tumor diameter was approximately 1.0 cm. Fifteen mice were randomly divided into 3 groups, treated with saline, HD or L-OHP +HD, respectively, once per 4 days (a total of 5 times). Saline and HD were administrated orally at a dose of 2.5 mg/kg. L-OHP was administrated *via* intraperitoneal injection at a dose of 8 mg/kg. The length and width (mm) of the tumor were measured every 3 days since the first intervention to calculate the tumor size. Tumor size (mm3) = 1/2 × length × width^2. All mice were sacrificed after the final measurement, with tumors stripped and weighed. Western blot test was performed to detect the expressions of PI3K, Akt, p-PI3K, p-Akt, MDR1/P-gp, GST-π, TOPOIIα, Survivin, and Bcl-2.


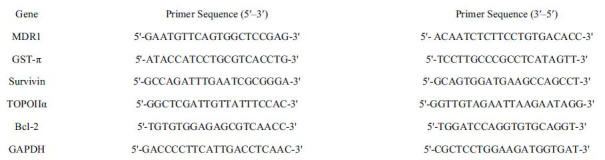


### 3.4 Statistical Analysis

Experimental data were analyzed using SPSS19.0 software. T-test and analysis of variance (ANOVA) were used to distinguish intergroup differences. *p* value <0.05 (two sides) was considered as statistical significance.

## 4 RESULTS

### 4.1 The Chemical Formula and the Potential Target of HD

A total of 267 potential genes of HD were obtained by summarizing and removing duplicate genes based on the TCMSP database, pharmMapper, SwissTargetPrediction, and HERB. A total of 3713 target genes for GC were acquired from OMIM, GeneCards and TTD. The target genes of L-OHP were obtained from GeneCards. A total of 1109 target genes were obtained. The potential genes of HD, GC, and L-OHP were, respectively, introduced into Venny 2.1.0 software to obtain the intersection gene. The result is shown in Fig. (**1**). In GC therapy, 55 targets intersecting three target sets are considered potential targets for HD mitigation of O-LHP resistance.

### 4.2 Construction of Protein Interaction Network and Screening of Core Genes

**Fig. (1):**
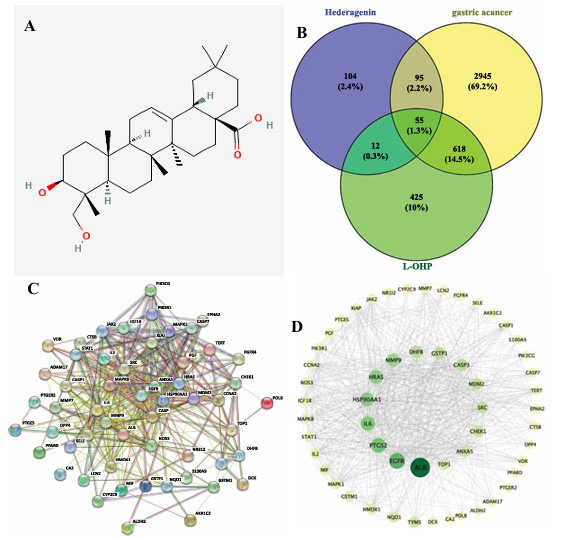
Screen disease and drug targets and topology analyses. **A-D)** Molecular structure of HD and Venn diagram for the obtaining the intersection gene of HD, GC and L-OHP. **C)** Using STRING database to construct PPI network. **D)** Visual analysis of PPI network.

In order to further explore the interaction mechanism of the cross-targets, 55 cross-targets were entered into the STRING database using the “multiple proteins” function. The species set as “Homo sapiens” yielded a protein interaction network (PPI) of 55 nodes and 417 edges (Fig. **1**). The network data was stored in TSV format and entered into CytoScape software for core gene analysis. The “CytoHubba” function of CytoScape software was used to calculate three topological parameters: Betweenness, MCC, and MNC. The first 20 genes were used and intersected, and 12 core targets were obtained, including ALB, CASP3, ANXA5, HSP90AA1, MMP9, EGFR, IL6, SRC PTGS2, HRAS, MAPK1, and MDM2.

### 4.3 GO Enrichment and KEGG Pathway Enrichment Analyses

To explore the biological functions of 12 core genes, GO enrichment and KEGG pathway enrichment analyses were performed in the DAVID database. The results of GO and KEGG enrichment are shown in Fig. (**2**).

### 4.4 Sensitivity of GC Tissue and Cell Lines to L-OHP

**Fig. (3):**
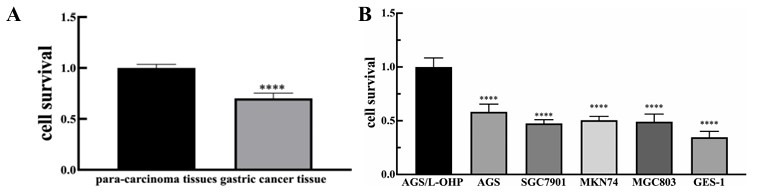
Chemosensitivity to L-OHP in GC tissues, para-carcinoma tissues and gastric cell lines. **A**) Clinical samples of GC and para-carcinoma tissues (L-OHP group), also different cell lines were collected. After L-OHP (5mg/L) administration, cellular survival was tested with MTT assay. Result of clinical tissues demonstrated that cellular survival in GC tissues was significantly lower than that in para-carcinoma tissues. **B**) As to cell lines, highest cellular survival was detected in AGS/L-OHP after L-OHP treatment. ****p < 0.0001 *versus* para-carcinoma tissues or AGS/L-OHP cells.

The cellular activity of GC tissue was significantly lower than that in the normal adjacent tissues after L-OHP intervention (*p* <0.01) (Fig. **3A**). Significantly lower activities were also observed for 4 kinds of GC cell lines as well as the gastric epithelial cell line GES-1 after using L-OHP compared to the cell line resistant to L-OHP, *i.e*., AGS/L-OHP (Fig. **3B**). In subsequent experiments, AGS/L-OHP cells with the strongest resistance to L-OHP were selected for study.

### 4.5 Effects of HD and HD+L-OHP on AGS/L-OHP Cell Line

**Fig. (4):**
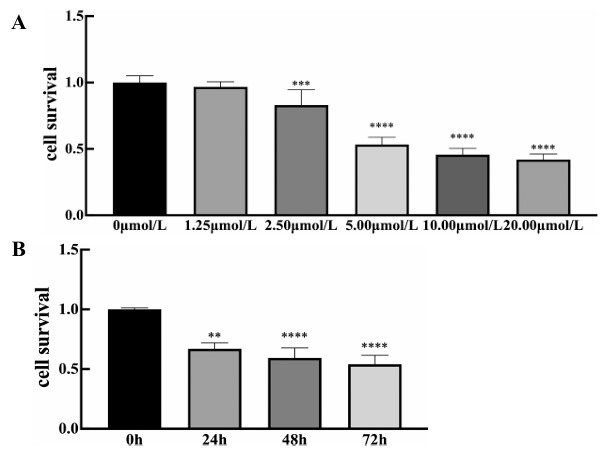
Effect of HD on cellular survival of AGS/L-OHP cells. **A**) Different concentrations were added to AGS/L-OHP cells. After 24h, cellular survival of different groups was detected with MTT assay. Cellular survival was significantly decreased in HD-intervention group in a dose-dependent fashion. **B**) HD could inhibit cellular survival of AGS/L-OHP cells in a time- dependent fashion. ***p <0.005 ****p < 0.0001 *versus* 0 μmol/L group or 0 hour.

AGS/L-OHP cells were treated using different concentrations of HD (*i.e*., 0, 1.25, 2.50, 5.00, 10.00, and 20.00 µmol/L). Compared to the L-OHP group, the activities of AGS/L-OHP cells decreased dose-dependently 24 hours after the intervention (Fig. **4A**). As shown in Fig. (**4B**), after HD intervention (10µmol/L), the activity of AGS/L-OHP cells declined temporally. In the present study, HD (10 µmol/L) at 24h served as a condition for further research *in vitro*.

**Fig. (6):**
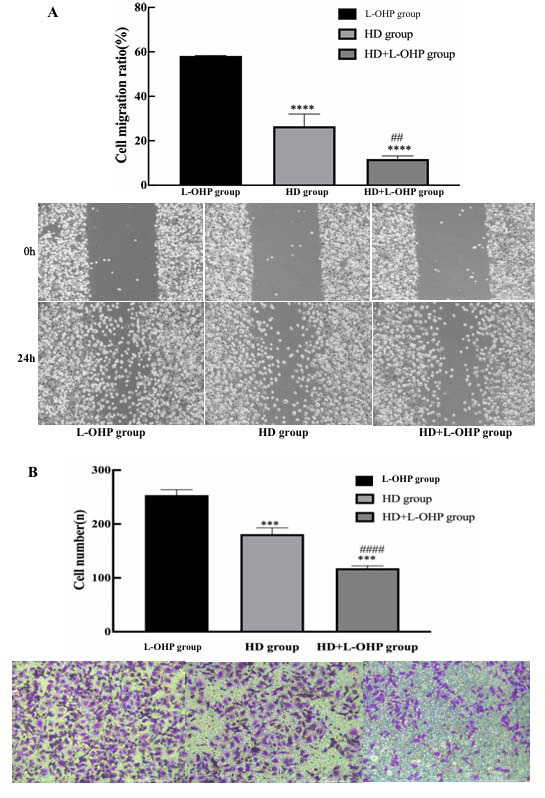
Effect of HD combined with L-OHP on migration and invasion of AGS/L-OHP cells. **A**) Results revealed that after HD or HD+L-OHP administration, migration of AGS/L-OHP cells decreased compared with L-OHP group, and migration of AGS/L-OHP cells decreased more obviously in HD+L-OHP group than in HD group. **B**) Similar results of AGS/L-OHP cells’ invasion were demonstrated in Transwell Chamber assay. ***p <0.005 *versus* L-OHP group; ^####^*p* <0.0001 *versus* HD group.

Fig. (**5**) shows the temporal changes in cellular activities across groups of L-OHP, HD intervention, and HD+L-OHP intervention. Compared to another two groups, the activity of AGS/L-OHP cells decreased most significantly over time for the HD+L-OHP group (*p* <0.01). Results of the Wound assay showed that the migration of AGS/L-OHP cells was lowest in the HD+L-OHP group, followed by the HD group and L-OHP group (*p* <0.01, Fig. **6A**). Similar results were observed in the Transwell assay (Fig. **6B**).

**Fig. (7):**
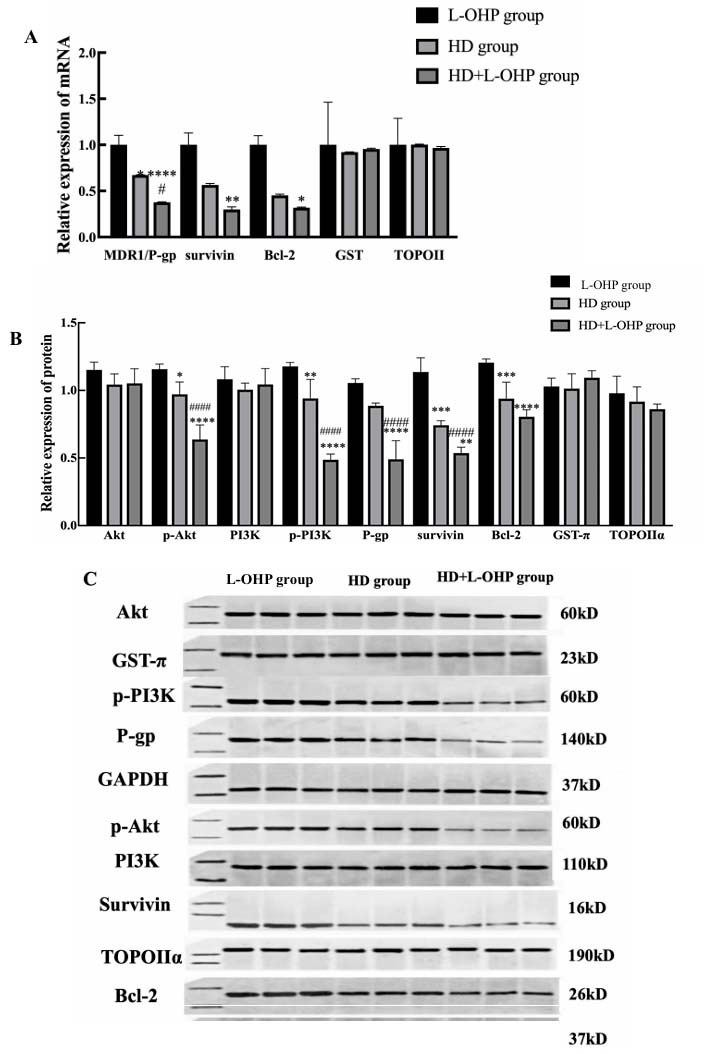
Effect of HD or HD+L-OHP administration on expression of MDR1/P-gp, GST-π, TOPOIIα, Survivin Bcl-2 and/or Akt, p-Akt, PI3K, p-PI3K in AGS/L-OHP cells. **A**) After treated with HD or HD+L-OHP, expression of MDR1/P-gp, Survivin and Bcl-2 in AGS/L-OHP decreased obviously, and in HD+L-OHP group, changes of these genes varied more evident than in HD group, as results of qPCR. **B** and **C**) Results of Western Blot assay. *p <0.05 *versus* L-OHP group; ^#^*p* <0.05 *versus* HD group.

The results of real-time RT-PCR (Fig. **7A**) and Western Blot (Fig. **7B**) showed that expressions of p-PI3K, p-Akt, MDR1/P-gp, Survivin, and Bcl-2 were significantly lower in AGS/L-OHP cells after HD or HD+L-OHP treatment, compared to the L-OHP group (*p* <0.01). However, there were no substantial inter-group differences in the expressions of GST-π and TOPOIIα.

### 4.6 Effects of HD and HD+ L-OHP on Nude Mice Models

**Fig. (8):**
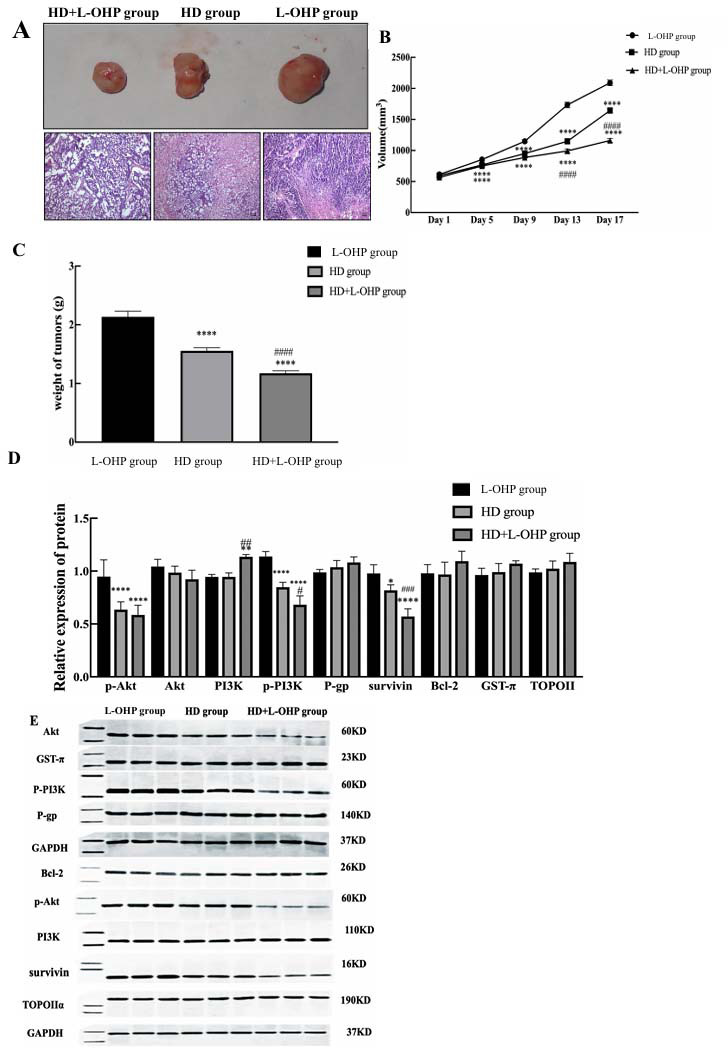
Effect of HD or HD+L-OHP intervention on tumors of nude mice transplanted with AGS/L-OHP cells subcutaneously. **A**-**C**) After HD or HD+L-OHP administration, it was confirmed that lowest tumor volume and weight was in HD+L-OHP group, and tumor volume and weight in HD group were lower than that in L-OHP group (*p* <0.01). **D** and **E**) p-Akt, p-PI3K, Survivin in tumor tissues of nude mice had the highest expression in L-OHP group, followed by HD group and HD+L-OHP group (*p* <0.01). ****p <0.0001 *versus* L-OHP group; ^####^*p* <0.0001 *versus* HD group.

The tumors from different nude mice models with their H.E staining are shown in Fig. (**8A**). The sizes and weights of tumors decreased from the highest in the L-OHP group to the lowest in the HD+L-OHP group (Figs. **8B** and **C**). The expressions of p-PI3K, p-Akt, and Survivin were highest in the tumor tissues of the L-OHP group and lowest in the HD+L-OHP group (Fig. **8D**). There were no substantial inter-group differences in the expressions of other proteins.

## 5 DISCUSSION

Despite the rapid development of healthcare management, the prognosis of patients with GC is still relatively unsatisfactory [16-19]. Chemotherapy is an important part of the treatment of GC. However, primary or acquired resistance of GC cells to drugs often leads to the failure of chemotherapy and thus triggers the recurrence and metastasis of tumors [20]. L-OHP is one dominant drug for GC treatment compared to the other first-line chemotherapeutic medicines, such as XELOXC (apecitabine + oxaliplatin) and SOX (Ticeo + oxaliplatin). L-OHP works by producing derivatives of hydration and intra-strand and inter-strand crosslinks, which inhibit DNA synthesis and thus cause cytotoxicity and anti-tumor activity [21]. However, GC cells may still exhibit resistance to L-OHP [22-24]. Our results indicated that the cell survival of AGS/L-OHP cells was higher than other GC cells after L-OHP intervention.

Recent studies have shown that HD has anti-tumor effects in cancer [25, 26]. Fang *et al.* reported that HD could inhibit the viability and promote apoptosis of cervical cancer cells [27]. The results of our subsequent *in vitro* and *in vivo* experiments indicated that the sensitivity of tumor cells to L-OHP increased significantly after HD intervention. Our results also showed that HD inhibited the migration and invasion of AGS/L-OHP cells. These findings suggest that HD may enhance the effect of L-OHP on GC cells, recommending the combined use of L-OHP and HD to improve chemotherapy. Moreover, our *in vitro* study found that the resistance of drug-resistance GC cells to apoptosis was also enhanced. Then, we detected the expressions of MDR1/P-gp, GST-π, TOPOIIα, Survivin, and Bcl-2 genes in AGS/L-OHP cells treated with HD and/or L-OHP. Our results showed that after HD intervention, expressions of MDR1/P-gp, Survivin, and Bcl-2 significantly decreased. The result indicated that the resistance of the GC cell line to L-OHP was also associated with the over-expression of apoptosis-associated proteins (*e.g.,* Bcl-2 and Survivin) and classic resistance proteins (*e.g.,* P-gp) and HD may be involved in the MDR of GC cells by inhibiting the expressions of MDR1/P-gp, Survivin, and Bcl-2.

To improve the effect of chemotherapy, it is necessary to explore the mechanisms underpinning the resistance of GC cells to L-OHP as well as the way to reverse it. The pathways mediating the resistance of GC cells to L-OHP could be very complex. Previous studies have reported that drug pump protein, detoxification pathways, and DNA repair patterns may contribute to the resistance of platinum-based agents, whose antineoplastic mechanism is mainly to induce apoptosis. The PI3K/Akt signaling pathway is considered to be a classic pro-cancer and pro-inflammatory pathway, which is generally highly expressed in human tumors. In normal tissues, PI3K/Akt signal transduction is normally activated, and in abnormal tissues, it can accelerate the malignant biological behavior of tumor cells and mediate tumorigenesis [28, 29]. In this study, core genes were screened through network pharmacology, and KEGG enrichment analysis was performed on the core genes. KEGG analysis showed that the PI3K-Akt signaling pathway containing more enriched genes may play a greater role in anti-gastric cancer. Xu *et al.* [30] reported that Nar promotes apoptosis in gastric cancer cells by blocking the PI3K/Akt signaling pathway and activating autophagy. Zhou *et al*. demonstrated that MYR induces the expression of Bax, Caspase-3, and Caspase-9 proteins by inhibiting the PI3K/Akt signaling pathway, thereby promoting the apoptosis of gastric cancer cells [31]. To clarify the mechanism of HD in affecting the resistance of GC cells to L-OHP, we detected the expressions of p-PI3K, p-Akt, Akt, and PI3K genes in AGS/L-OHP cells treated with HD and/or L-OHP. Our results showed that after HD intervention, expressions of p-PI3K and p-Akt significantly decreased. This suggests that HD may reduce the resistance of AGS/L-OHP cells to L-OHP by inhibiting the expressions of the PI3K/Akt signaling pathway.

## CONCLUSION

In summary, our study observed significant resistance of GC cells to L-OHP, which could be effectively alleviated by HD. The effect of HD may be associated with suppressing the expressions of the PI3K/Akt signaling pathway. HD may relieve the resistance of GC to L-OHP. However, the specific molecular mechanisms should be fully explored. In the treatment of GC, we recommend the combined use of HD and L-OHP to improve the effect of chemotherapy. However, this study only focused on the pathways and mechanisms, and core targets were not validated in this study, which needs to be validated in future studies. Moreover, large clinical studies are also necessary to confirm our findings.

## Figures and Tables

**Fig. (2):**
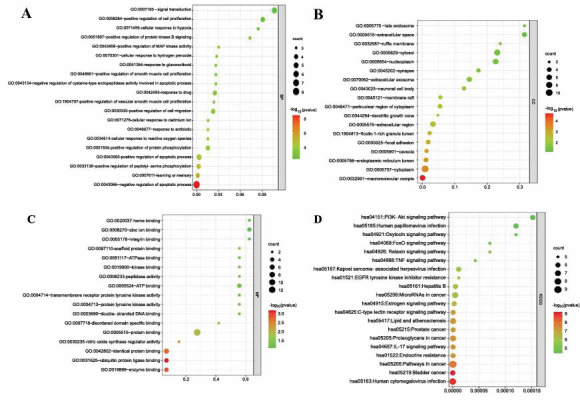
GO enrichment and KEGG pathway enrichment analyses. GO enrichment analysis bar chart, KEGG enrichment analysis bar chart.

**Fig. (5):**
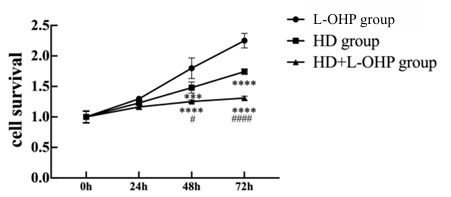
Effect of HD combined with L-OHP on cellular activity of AGS/L-OHP cells. The activity of AGS/L-OHP cells treated with HD+L-OHP was significantly lower than cells treated with HD or L-OHP group. ****p <0.01 *versus* L-OHP group, ^####^*p* <0.0001 *versus* HD group.

## Data Availability

The data of current study are available from corresponding author on a reasonable request.

## LIST OF ABBREVIATIONS

GC: Gastric Cancer
HD: Hederagenin
L-OHP: Oxaliplatin
PPI: Protein-Protein Interaction

## STANDARDS OF REPORTING

STROBE guidelines and methodology were followed.
